# Non-radioactive and sensitive tracking of neutrophils towards inflammation using antibody functionalized magnetic particle imaging tracers

**DOI:** 10.7150/ntno.50721

**Published:** 2021-02-12

**Authors:** Prashant Chandrasekharan, K.L. Barry Fung, Xinyi Y. Zhou, Weiwen Cui, Caylin Colson, David Mai, Kenneth Jeffris, Quincy Huynh, Chinmoy Saayujya, Leyla Kabuli, Benjamin Fellows, Yao Lu, Elaine Yu, Zhi Wei Tay, Bo Zheng, Lawrence Fong, Steven M. Conolly

**Affiliations:** 1 Department of Bioengineering, University of California, Berkeley, California 94720, United States.; 2 UC Berkeley-UCSF Graduate Group in Bioengineering, California, United States.; 3 Department of Electrical Engineering and Computer Sciences, University of California, Berkeley, California 94720, United States.; 4 UCSF Helen Diller Family Comprehensive Cancer Center, University of California, San Francisco, California 94143, United States.

**Keywords:** magnetic particle imaging, white blood cells, antibody, medical imaging, inflammation, superparamagnetic iron oxide nanoparticles

## Abstract

White blood cells (WBCs) are a key component of the mammalian immune system and play an essential role in surveillance, defense, and adaptation against foreign pathogens. Apart from their roles in the active combat of infection and the development of adaptive immunity, immune cells are also involved in tumor development and metastasis. Antibody-based therapeutics have been developed to regulate (i.e. selectively activate or inhibit immune function) and harness immune cells to fight malignancy. Alternatively, non-invasive tracking of WBC distribution can diagnose inflammation, infection, fevers of unknown origin (FUOs), and cancer. Magnetic Particle Imaging (MPI) is a non-invasive, non-radioactive, and sensitive medical imaging technique that uses safe superparamagnetic iron oxide nanoparticles (SPIOs) as tracers. MPI has previously been shown to track therapeutic stem cells for over 87 days with a ~200 cell detection limit. In the current work, we utilized antibody-conjugated SPIOs specific to neutrophils for *in situ* labeling, and non-invasive and radiation-free tracking of these inflammatory cells to sites of infection and inflammation in an *in vivo* murine model of lipopolysaccharide-induced myositis. MPI showed sensitive detection of inflammation with a contrast-to-noise ratio of ~8-13.

## Introduction

**Figure 1:**
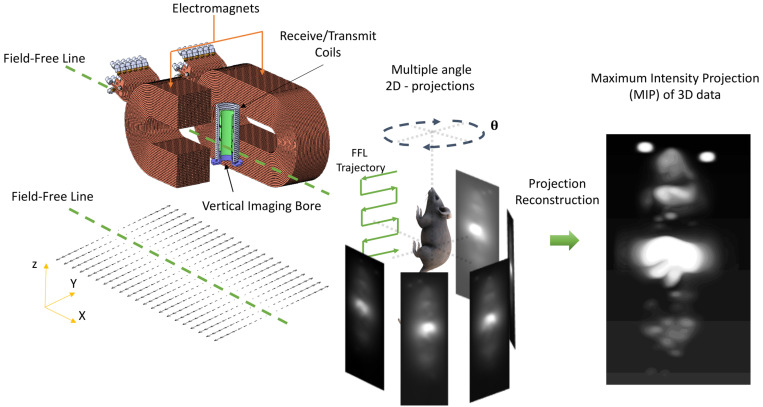
** Magnetic Particle Imaging** using a custom-built vertical bore 6.3 T/m Field- Free Line (FFL) scanner. The MPI scanner allows for 2D projection and 3D tomographic imaging of the spatial distribution of superparamagnetic iron oxide nanoparticles tracers (SPIOs). SPIOs obey Langevin physics; there is high magnetic saturation in response to an applied field and zero coercivity or remanence once the field is removed. In MPI, a time- varying field is applied and only the particles at the FFL flip in response. The flip generates a signal in a receiver coil due to Faraday's law of induction, and rastering the FFL allows the signal to be spatially localized. The FFL scanner can acquire a single 2D projection, or multiple 2D projections at various angles. Classical projection reconstruction algorithms can be implemented to reconstruct 3D MPI images from 2D projections. (2D: two-dimension; 3D: three-dimension; FFL: field-free line; MIP: maximum intensity projection; SPIO: superparamagnetic iron oxide nanoparticles).

Magnetic particle imaging (MPI) is an emerging non-invasive tracer imaging modality that images superparamagnetic iron oxide nanoparticle tracers (SPIOs) using their non-linear magnetization response (see Figure 1). The safety profile of the MPI tracer makes it suitable for use even in pediatric patients and those suffering with chronic kidney disease. Long-circulating MPI tracers facilitate imaging of the circulatory system and provide valuable information on pathologies associated with vascular defects such as in cancer 1, lower gastrointestinal abdominal bleed 2, stroke 3, traumatic brain injury 4, and diseases involving lung ventilation and perfusion 5, 6. MPI also allows for the monitoring of chemotherapy 7. Because the SPIO tracer in MPI generates high contrast images with long persistence 8, interest in MPI cell tracking has grown, especially for assessing the fate of cell-based therapeutics such as stem cell therapies and immune cell therapies 9-14.

MPI signal is known to be highly sensitive and linearly quantitative, including when other entities of interest, such as cells, are attached to SPIOs. Notably, a detection sensitivity of ~200 cells was reported in one of the seminal cell tracking studies using MPI 15.The signal from MPI tracers does not decay over time and persists until the tracer is biochemically hydrolyzed. This has allowed for MPI studies that track SPIO-labeled stem cells for over ~87 days to monitor the long-term fate of therapeutic cells 15, 16. The combination of exceptional sensitivity and capacity for long-term imaging is why MPI is particularly well-suited to tracking cell therapeutics, in which cells are known to persist for months or even years 17-19. MPI has also been used to probe for visceral surgical sites, for functional grafting of islet cells in a preclinical model 20, and for monitoring successful islet cellular graft implantation and insulin production. MPI also has a superior dose limited-sensitivity - in a recent study, 2D MPI projection scans using a clinically relevant dose of 5 mg of Fe/kg (as was used in this work) were sufficient to detect GI bleed with rates as low as 1 and 5 µl/min 2.

The role of immune cells has been implicated in the onset of various pathologies and the inflammatory process has thus been observed during the early stages of infection, cancer, and neurodegenerative diseases 18, 21-23. The tracking of immune cells and their responses are thus invaluable for early diagnosis of such pathologies.

There are a number of approaches for imaging inflammation and infection 24. Anatomical imaging methods are used in monitoring morphological changes in organs, especially in bones, tendons, muscles, and certain soft tissues. X-ray Computed Tomography (CT) and magnetic resonance imaging (MRI) are often used for detecting changes in bones, tendons, and some soft tissues, while ultrasound is used for assessing the effects of infection and inflammation in dermal and soft tissue 24.

Functional imaging, using nuclear medicine techniques, allows for a more comprehensive diagnosis of functional tissue impairment due to infection and inflammation and provides a much earlier diagnosis before the morphology is altered. These methods detect the onset of inflammation, characterized by vessel dilation, changes to extracellular spaces, and most notably, increased accumulation of neutrophils and other phagocytic immune cells (e.g. monocytes and macrophages) 25, 26.

Neutrophils are the first responders in innate immunity (which forms the initial response of the body to infection), and their role is to kill the source of infection and modulate both the innate and adaptive immune response. Tracking neutrophils are therefore an ideal functional imaging method that would enable the early detection of inflammation.

Functional imaging often relies on tracer imaging modalities, which predominantly image the biochemistry of infection and inflammation. The Ga-67 tracer used in Gallium citrate scintigraphy, for instance, binds to transferrin receptors (CD71) that are highly expressed by inflammatory cells. Ga-67 is also known to bind directly to siderophore molecules of bacteria, enabling the direct visualization of the sites of infection 27, 28. In another approach, In-111 tagged Somatostatin receptor (SSTR) binding analogues were used to identify granulomatous and chronic inflammation that had an over-expression of SSTR 29, 30. Recently, inflammation-associated enzymatically responsive MPI tracer was developed to image vulnerable atherosclerotic plaque 31.

Direct labeling and tracking of immune cells with tracers is another option for tracking inflammation and infection. White blood cell (WBC) tracking traditionally refers to the clinical method utilizing scintigraphy or single photon emission computed tomography for diagnosis of inflammation and infection, and is often called for in cases of fever of unknown origin (FUO) 32, 33. WBC tracking is achieved by either *ex vivo* tracer labeling of immune cells (isolated from a blood draw) which are re-introduced via intravenous injection (*i.v.*), or by *in situ* tracer labeling of immune cells. *Ex vivo* labeling of cells can be achieved chemically by precipitating the tracer within the immune cells of interest 34, 35 or biologically by allowing the isolated immune cells to phagocytose the tracer 36-38. Recently, new and exciting approaches for *ex vivo* labeling and tracking of therapeutic T-cells 39 and macrophages 40, 41 using MPI have also been reported.

While *ex vivo* labeling of tracers is well established and commonly done clinically, the technique is rather elaborate and requires long preparation times and expertise. In comparison, *in situ* cell labeling is performed by injecting the tracer directly into the subject and utilizing innate tracer properties and pathophysiology to provide specificity, and thus is a versatile approach that preserves labeled cell function and viability. Examples of this technique include using antibody-labeled tracers that target specific surface proteins expressed by granulocytes. For instance, antibodies that selectively bind to “non-specific cross-reacting antigen” 90 and 95 (NCA-90 and NCA-95) are being tagged with Tc99m or In-111 radioisotopes for scintigraphy 42.

Nano-sized colloids are also used in *in situ* labelling for imaging inflammation. Tc99m- albumin nanocolloids, Tc99m-sulfur colloids and iron oxide nanoparticles are readily taken up by the organs of the reticuloendothelial system (RES), including the liver, spleen, and bone marrow. The non-specific extravasation of the nano-colloids at sites of inflammation is considered the general mechanism of accumulation for these tracers 43. Recent research points to a more specific mechanism in which *i.v.* administered nanocolloids are specifically taken up by polarized phagocytes in the RES system that then accumulate at sites of inflammation. This mechanism for imaging inflammation has been thoroughly vetted for MRI 44 by using different colloidal tracers of iron oxide nanoparticles 45-47, and 19F 48-50.

In the current work, we report an approach for *in situ* labeling and tracking of granulocytes/neutrophils to sites of lipopolysaccharide induced myositis using MPI. To achieve *in situ* labeling we selected an MPI tracer with surface antibodies of anti-Ly6G, which are specific towards surface antigens expressed on murine neutrophil immune cells. We characterized the MPI performance of these tracer using our home-built arbitrary wave relaxometer (AWR) 51 and scanner 2 and evaluated the specificity and sensitivity of these tracers *in vitro*. Finally, we demonstrated the *in vivo* homing of the labeled neutrophils to sites of myositis and validated the findings with histology.

## Results and Discussion

### MPI tracers for tracking WBCs

In the current work, we have utilized commercially available SPIO nanoparticles for tracking WBCs using MPI. Antibody-conjugated iron oxide nanoparticles (antibody-SPIOs) are extensively used to isolate a selective cell population from a mixed cell population 52. Antibody-SPIOs that bind to specific cell surface markers of a cell population can be “pulled” using magnetic fields. This method has high specificity for isolating cells and preserves the overall functionality of the cells. We identified commercially available anti-Ly6G SPIOs that selectively tag Ly6G antigen expressed on murine neutrophils *in situ* and used VivoTrax*^TM^* as a control. The properties of VivoTrax*^TM^* and anti-Ly6G antibody bound tracer are summarized in Tables 1 & 2.

**Figure 2:**
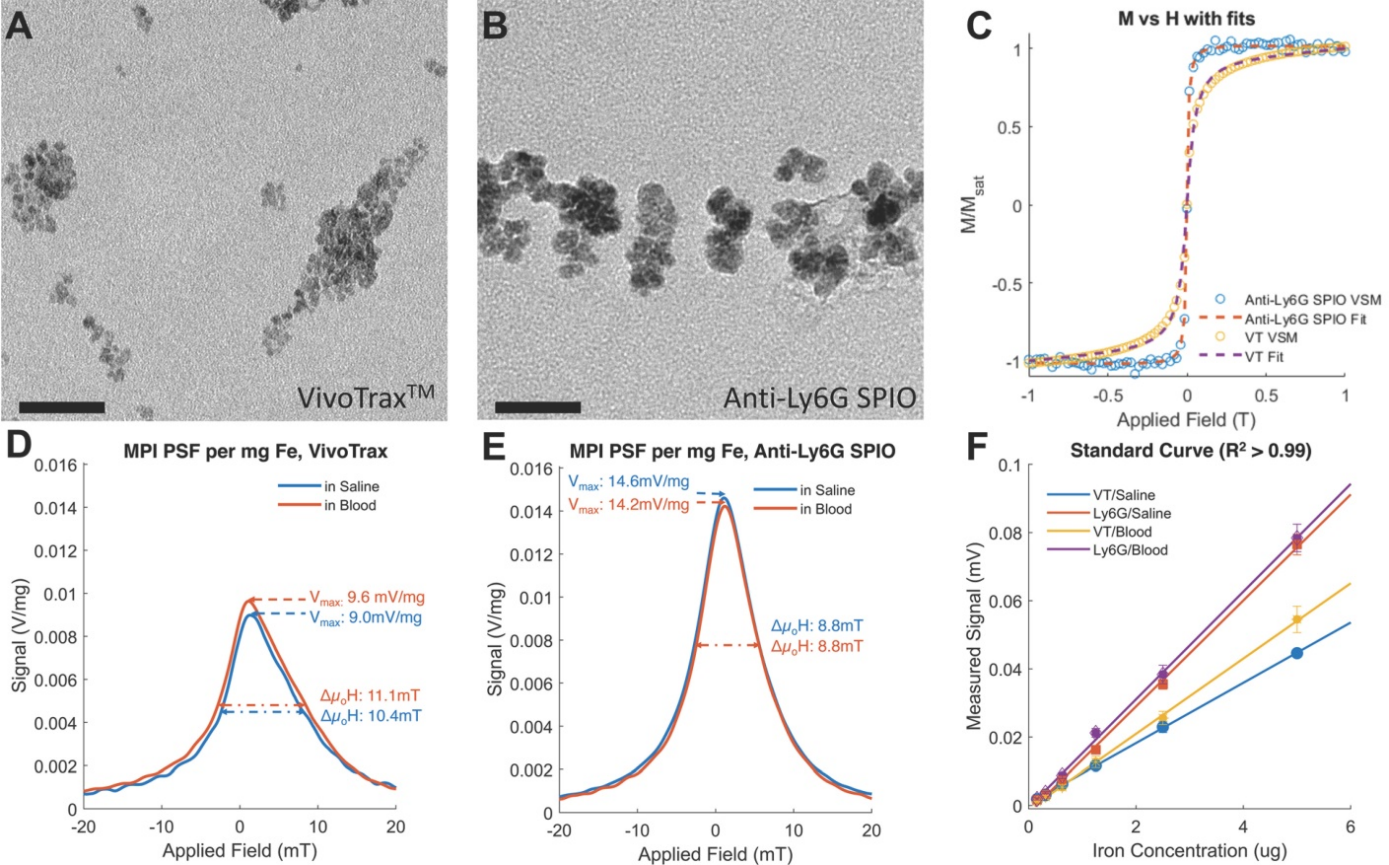
**Magnetic characterization of the anti-Ly6G SPIOs compared to VivoTrax***^TM^* **(VT)**. Representative transmission electron microscopy (TEM) images (scale bar = 50 nm) were taken of both **(A)** VT with core diameter = 5.4 ± 1 nm and **(B)** anti-Ly6G SPIOs with core diameter = 14 ± 2 nm. **(C)** The magnetization curve of VT and the anti-Ly6G SPIOs as measured in a VSM were fit to a log-normal diameter distribution. Anti-Ly6G SPIOs had a mean magnetic diameter (*D_v_*) of 14.8 nm, with a log-normal standard deviation (*σ*_ln d_) of 0.5. VT had (*D_v_*) = 9.8 nm and *σ* = 0.2. We used a custom arbitrary waveform relaxometer (AWR) to measure the MPI point spread function (PSF) of both samples in saline and in blood. *Representative* PSFs are shown in **(D)** for VT and **(E)** for the anti-Ly6G SPIOs. Note the signal change in the PSF of VT, with the change in signal being significant (*p <* 0.05). In comparison, anti-Ly6G SPIOs showed stable behavior in both solvents (change in signal was not significant (*p* = 0.7 *>* 0.05). **(F)** A standard curve of MPI signal was taken versus iron concentration for all particles in mouse blood and in saline, with mean and standard error given over *n* = 3 samples (with 8 acquisition repeats averaged per sample). Both particles show linearity with concentration in both solvents (*R*^2^ *>* 0.99). The anti-Ly6G SPIOs showed significantly better resolution (*p <* 0.01) and sensitivity (1*.*8 ± 0*.*3 times better, with *p <* 0.01) than VivoTrax*^TM^*. VivoTrax*^TM^* showed a significant (*p <* 0.01) change in sensitivity (V/g) in different media, while the anti-Ly6G SPIOs did not (*p >* 0.05). This standard curve was also repeated in the scanner and is included in Figure 3. (H: magnetic field; M: Magnetization; MPI; magnetic particle imaging; PSF: point-spread function; SPIO: superparamagnetic iron oxide; VT: VivoTrax^TM^).

Both VivoTrax*^TM^* and anti-Ly6G SPIOs are synthesized by the co-precipitation of iron salts under a reducing environment, in a similar manner to numerous other iron nanoparticle supplements 53, and have a carboxydextran coating for colloidal stabilization in physiological fluids 52-54. VivoTrax*^TM^* is a carboxydextran cross-linked clustered iron oxide nanoparticle with an average core diameter of 5.4 nm and a hydrodynamic diameter of 61 nm. The anti-Ly6G SPIO is coated with dextran and functionalized with IgG1 monoclonal antibody, and also appeared clustered under transmission electron microscopy (TEM). They had an average core diameter of 14 nm and a hydrodynamic diameter of 78 nm. Figure 2 shows the TEM images, magnetic response, and MPI response of the tracers used in this study. Both VivoTrax*^TM^* and the anti-Ly6G- SPIOs were superparamagnetic based on the M-H curve measured using a vibrating sample magnetometer (VSM). Notably, the anti-Ly6G-SPIO had a smaller M*_sat_*, but a larger initial susceptibility (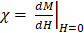) compared to VivoTrax*^TM^* 55, 56. The magnetic diameter and distribution were estimated by fitting the data to the ensemble Langevin response from a log-normal distribution of particles (akin to Chantrell's method) 57, 58. The non-linear least squares estimate for the magnetic diameter of the anti-Ly6G-SPIOs and VivoTrax*^TM^* were 14.8 nm (*σ*_ln d_ = 0.5) and 9.8 nm (*σ*_ln d_ = 0.2) respectively, showing good correspondence to the physical size estimate from TEM.

**Figure 3:**
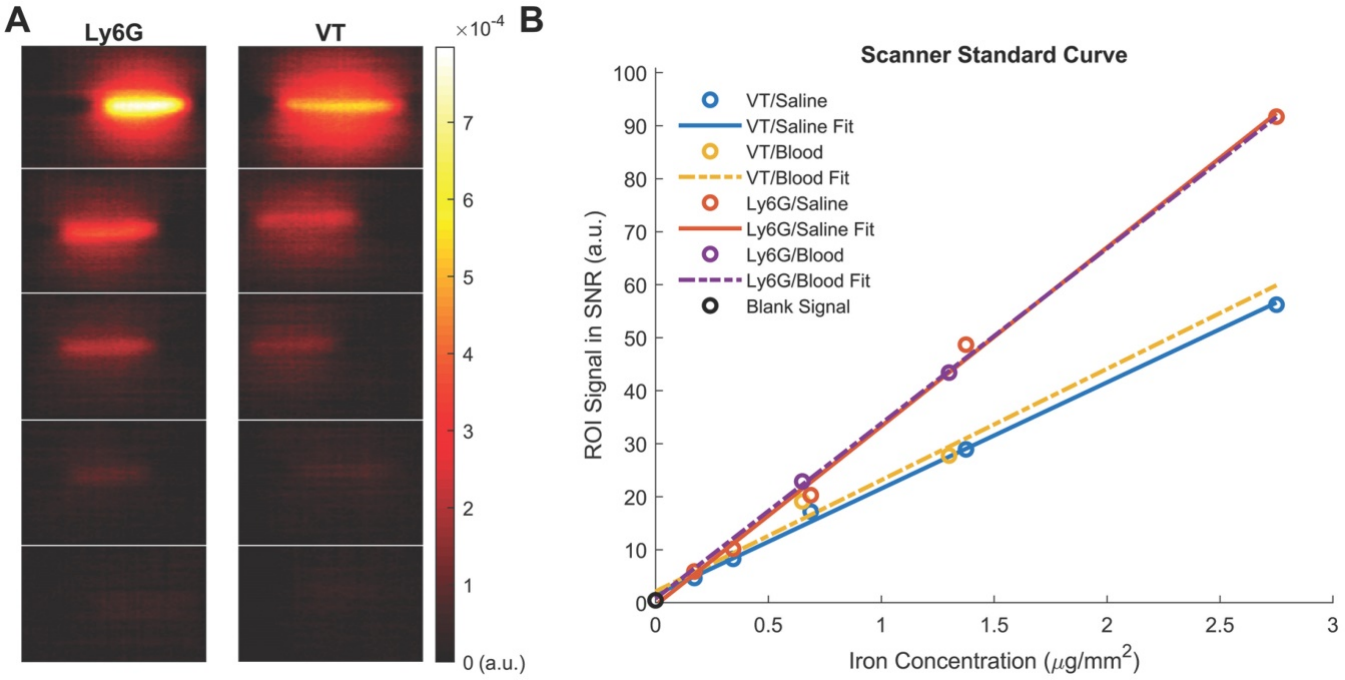
** Standard Curve of the anti-Ly6G SPIOs and VivoTrax***^TM^* **(VT) in our Field-Free Line Scanner**. (A) the images of the standard curve for anti-Ly6G SPIOs and VT in saline are shown in linear heat scale scaled to the maximum signal between both curves (FOV = 4.1 x 6.2 cm^2^, scan time = 37s). Note the increased conspicuity of anti-Ly6G SPIOs when compared to VivoTrax*^TM^* (B) The standard curves in saline (given as the sample SNR averaged from the 5 pixels by 5 pixels center of each sample), alongside standard curves in blood. The linear fits to each particle and solvent pair are mostly good (R^2^ *>* 0.99), except for VT in blood (R^2^ = 0.85) - this can be attributed to experimental error in the sample at 0.7 μg mm*^-^*^2^. Note the similarity to Figure 2F, with the sensitivity of the anti-Ly6G SPIOs being 1.7 ± 0.5 times better than that of VivoTrax*^TM^*. (SNR: signal-to-noise ratio; SPIO: superparamagnetic iron oxide; VT: VivoTrax^TM^).

We further tested the MPI performance of VivoTrax*^TM^* and anti-Ly6G SPIOs using our home-built arbitrary wave relaxometer (AWR) 51 and our scanner 2 in whole murine blood and in 1X phosphate buffered saline (PBS) (Figures 2 and 3). As *i.v.* administered tracers first partition within the blood before distributing to different parts of the body, we wanted to examine whether the MPI tracer properties were influenced by the presence of blood. We observed a 1.8 ± 0.3 times higher MPI signal per gram of iron from anti-Ly6G SPIOs compared to VivoTrax*^TM^* in 1X PBS in the AWR (based on the slope of standard curve in Figure 2F), and a 1*.*7 ± 0*.*5 times relative signal for similar samples in the scanner (Figure 3). Moreover, the anti-Ly6G SPIO had a better MPI resolution, equivalent to 1.26 mm (or 8.8 mT) in a 7 T/m MPI field gradient, whereas VivoTrax*^TM^* had a resolution of 1.49 mm (or 10.4 mT). The better performance in MPI signal and resolution of the anti-Ly6G SPIO compared to VivoTrax*^TM^* can be explained through the Langevin behavior of the nanoparticle tracer. In MPI, the magnetization of SPIOs is controlled by a time-varying sinusoidal magnetic field (typically, with 20 mT amplitude at 20 kHz). The resultant MPI signal scales with the rate of change of magnetization 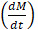
due to Faraday's law 59. The improved MPI signal for anti-Ly6G SPIOs as compared to VivoTrax*^TM^* can thus be attributed to its higher susceptibility near zero applied field (Figure 2C), which in turn may be explained by the anti-Ly6G SPIOs' larger magnetic core (*D_v_* in Table 2). The resolution of the MPI tracer depends on the width of the transition from positive to negative saturation (2*H_sat_*), which scales proportionally to 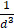 where *d* is the diameter of the nanoparticles. The larger core size of the anti-Ly6G SPIOs could thus explain the significant gain in resolution.

**Figure 4:**
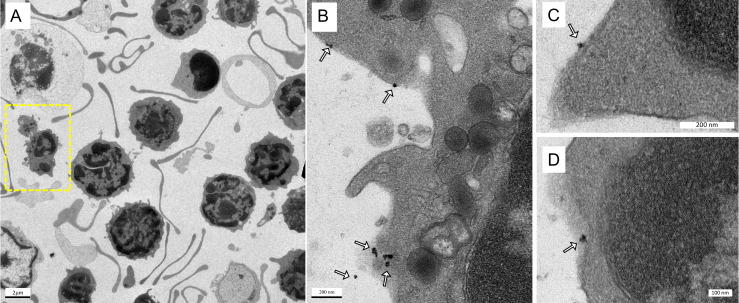
**Labeling of immune cells using anti-Ly6G SPIOs.** (A) TEM images of whole blood sample incubated with anti-Ly6G SPIOs (scale bar = 2 μm). (B), (C) Magnified TEM images (scale bar = 200 nm) & (D) (scale bar = 100 nm) of yellow box marked in (A). The arrows point to the blood cell-membrane bound nanoparticles. The membrane bound particles had a core diameter of 16 ± 5 nm, corresponding well with the size of anti-Ly6G SPIO particles observed in Figure 2 (SPIO: superparamagnetic iron oxide (nanoparticles); TEM: transmission electron microscopy).

We also observed that the peak signal of VivoTrax*^TM^* (Figures 2D & F) in 1X PBS was different compared to its properties in whole blood. In contrast, the properties of anti-Ly6G SPIOs remained the same (Figures 2E & F). The change in behavior arises from the particle interaction with the blood sample. Note that the particles are well suspended as colloids, so there is minimal interparticle interaction. The anti-Ly6G SPIOs interact with specific cell surface antigens (as shown in Figures 4A-D), while carboxydextran-coated SPIO particles such as VivoTrax*^TM^* are phagocytosed by the blood cells and aggregated within endocytic vesicles 37. A dipole-dipole interaction within the vesicles could alter the effective susceptibility 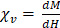 due to change in fractional volume of the particles. These subtle changes observed using the AWR were less pronounced in the standard curve taken in the scanner (Figure 3B). This could be due to inherent differences in sensitivity between a 1D-arbitrary wave relaxometer versus an image scanner, experimental errors, as well as the effects of relaxation induced blurring on a non-point source phantom 51, 59, 60. Signal changes observed using VivoTrax*^TM^* in AWR are being evaluated for use with pulsed and color MPI for studying the influence of micro-environment on the particles 51,61-64. We ourselves have observed similar changes in our previous publication with VivoTrax*^TM^* labeled stem cells and believe that these observations warrant further investigation 15. In either case, the invariability in media and improved resolution of the commercially available anti-Ly6G SPIOs used for magnetic cell sorting make them competitive for MPI imaging.

### Cell Enrichment Analysis of MPI Tracers

Next, we assessed the binding and enrichment efficiency of the MPI tracers to neutrophils using two methods, a qualitative assessment using TEM (Figure 4) and a quantitative approach using flow cytometry (Figure 11). Note that the proportions of cell types in healthy human and mouse blood are different; for example, a healthy human has 50-70% peripheral blood neutrophils 65 compared to only 20-30% neutrophils in murine subjects 66. Here, we tested the enrichment efficiency of the anti-Ly6G SPIO tracer in selectively binding and enriching the WBC of interest (i.e. neutrophils, which form the largest subset of granulocytes). The anti-Ly6G antibody is a monoclonal IgG antibody with a high binding specificity towards the antigen or an epitope (antigen binding site), with a binding constant typically in the order of nMs 67.

Figures 4A-D show the TEM images of anti-Ly6G SPIOs incubated in murine whole blood sample. Of the various WBC cell populations found in the sample, only a few cells were found to be tagged with anti-Ly6G SPIOs. The anti-Ly6G SPIOs were predominantly membrane bound and were not found in the cytoplasm of the immune cells. The core diameter of the particles in the TEM were 16 ± 5 nm and corresponded well in size with the TEM of particles in Figure 2. Furthermore, the particle distribution in the fixed sample in Figure 4 corroborates well with previously published research articles 68, 69. Using flow cytometry, we then evaluated differential population enrichment of WBCs using three SPIOs: (1) anti-Ly6G SPIOs, (2) anti-F4/80 SPIOs and (3) VivoTrax*^TM^*.

**Figure 5:**
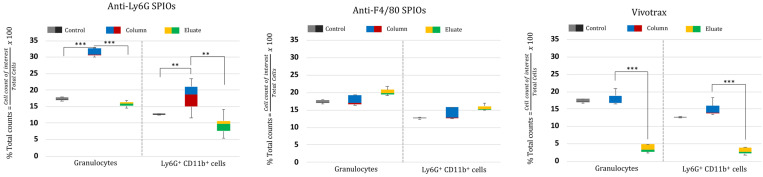
**Cell enrichment analysis of MPI tracers using flow cytometry:** Flow cytometry data represented as % enriched immune cell population (normalized to total cells counted) in the “Eluate” and “Column” content collected during and after magnetic separation of RBC lysed mouse blood using anti-Ly6G SPIOs, anti-F4/80 SPIOs and VivoTrax*^TM^*. The control data represents the % of immune cells of interest (normalized to total cells counted) in unseparated RBC lysed blood samples. We were particularly interested in the granulocyte population (of which neutrophils are the largest subset) and the CD11b^+^ & Ly6G^+^ population. A significant enrichment of both granulocyte population and CD11b^+^ & Ly6G^+^ was observed in the “Column” using anti-Ly6G SPIOs. Anti-F4/80 SPIOs showed no enrichment of granulocytes or neutrophil population, which is expected due to lack of specificity to those populations. Finally, Vivotrax*^TM^* showed significant enrichment of both granulocytes and neutrophil population (P*<*0.05 (**) and P*<*0.001 (***)) but the percentage relative enrichment in the “column” was not significant with respect to control (P*>* 0*.*05) (MPI: magnetic particle imaging; RBC: red blood cells; SPIO: superparamagnetic iron oxide).

The RBC-lysed blood samples were incubated with the SPIOs and run through a magnetic column once 52. Two populations were recovered from this column: the “eluate” of cells collected from the column under a magnetic field (i.e. cells not bound to SPIOs that can be eluted through the column) and the remaining cells recovered from the “column” (i.e. after the initial eluate is recovered & the magnetic field is removed so that cells bound to SPIOs can be recovered). The “eluate” and “column” were analyzed using flow cytometry to determine the number of granulocytes and CD11b^+^ & Ly6G^+^ cell types relative to the total number of cells. Granulocytes and CD11b^+^ & Ly6G^+^ from untouched RBC-lysed blood samples served as a control providing a gauge of initial % immune cell population in the blood. Figure 5 shows the % total counts of granulocytes and CD11b^+^ & Ly6G^+^ WBCs from the “eluate” and “column” with relation to the control for each SPIO used. It should be noted that neutrophils are the largest subset of granulocytes, followed by eosinophils and basophils. Eosinophils are found to vary between 0-7% of total mouse WBCs, while basophils are almost negligible in mouse blood 66. Eosinophils do exhibit a varying lower expression of Ly6G marker on the cell surface 70. As such, gating the granulocyte population utilizing forward and side scattering profiles provides an upper bound to the neutrophil population, while counting the number of cells with CD11b^+^ & Ly6G^+^ gating provides a more specific estimate of neutrophils. As such, we evaluated the degree of enrichment of both granulocytes and neutrophils (i.e. CD11b^+^ & Ly6G^+^ cells) in the “column” for each SPIO under investigation relative to the degree of depletion of the same population in the “eluate”.

The anti-Ly6G SPIOs showed significant enrichment of both the granulocyte population and the CD11b^+^ & Ly6G^+^ population in the “column” relative to the “eluate”. In comparison, anti-F4/80 SPIOs did not show enrichment of the granulocyte or neutrophil (CD11b^+^ & Ly6G^+^) population (eluate vs. column in comparison to the control). F4/80 is a marker of tissue residual monocytes/macrophages and is sparingly expressed in circulating WBCs 71, 72. It is thus important that we did not notice enrichment of circulating granulocytes/neutrophils populations with anti-F4/80 SPIOs. Finally, VivoTrax*^TM^* showed significant change in the granulocyte (& neutrophil) population in the “column” compared to “eluate”. However, the relative population of granulocyte (& neutrophil) in “column” was similar to that of control.

Combined, the populations in “column” vs “eluate” for VivoTrax*^TM^* appear to indicate that the incubation resulted in the labeling of all WBC subtypes, as shown in the overall reduction in the WBC cell counts in the eluate from the magnetic column (Figure 5, VivoTrax*^TM^* Column vs. Eluate with respect to control). Though we did not acquire a TEM image of VivoTrax*^TM^* equivalent to that in Figure 4, there is a plethora of literature that has demonstrated that dextran-coated SPIOs are phagocytosed and internalized by peripheral blood cells 37, 46, 73-76. In fact, it was previously shown that *i.v.* administered VivoTrax*^TM^*is quickly taken up by the phagocytic cells of the RES system 54, that are distributed predominantly in the liver.

### *In vivo* evaluation of tracers

We evaluated the tracer biodistribution in a healthy mouse and a mouse model of myositis of the right thigh muscle induced by bacterial lipopolysaccharides (LPS). For *in vivo* evaluation we investigated the distributions of anti-Ly6G SPIOs and VivoTrax*^TM^* in mice. LPS derived from gram negative bacteria are known to cause localized inflammation with systemic elevation of circulating inflammatory blood cell count 77. Neutrophils are the first responders to inflammation and infection and are followed by a slow migration of monocytes that differentiate into mature macrophages, thus providing a suitable target for imaging acute inflammation.

**Figure 6:**
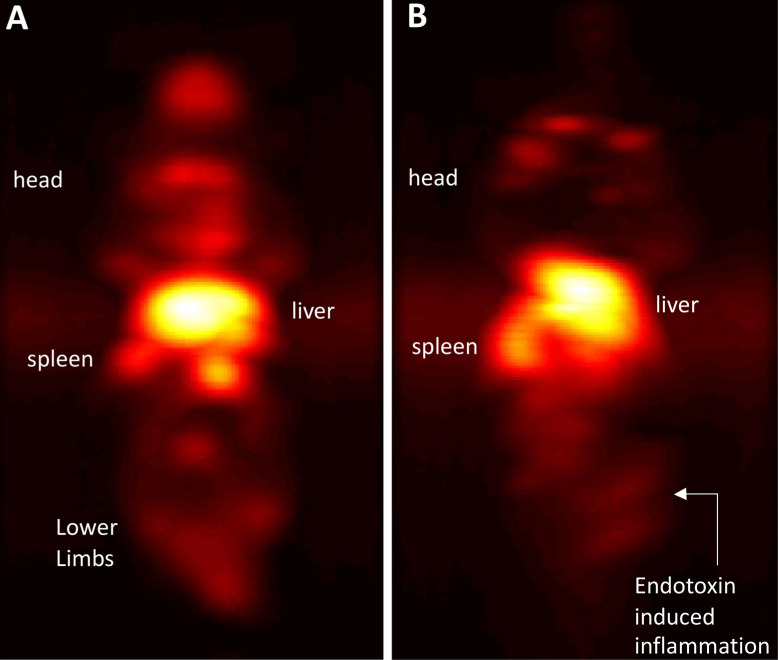
** Immuno-MPI**: Anterior-posterior maximum intensity projection (MIP) images of 3D MPI data, 24 hours post anti-Ly6G antibody SPIO tracer administration in (A) a healthy mouse (B) mouse with LPS endotoxin induced myositis in the right leg (MIP: maximum intensity projection; MPI: magnetic particle imaging; SPIO: superparamagnetic iron oxide).

**Figure 7:**
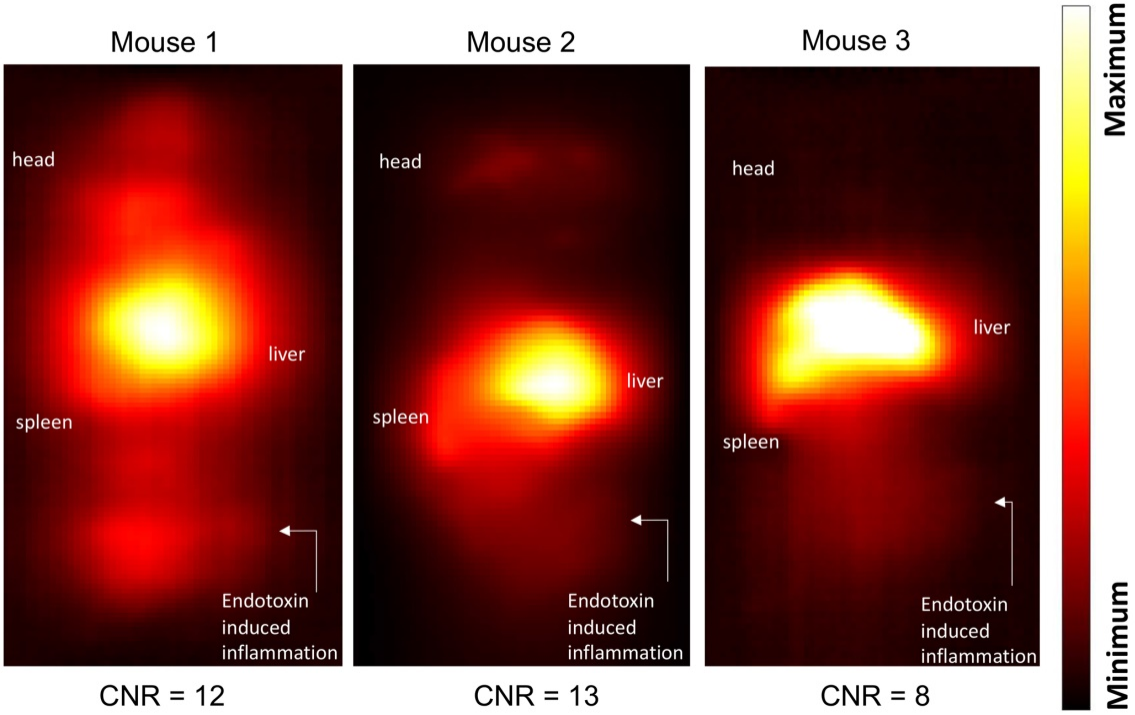
**Inflammation** images in three different mouse subjects with myositis and *i.v.* administered **anti-Ly6G-SPIO**. Mouse 1 had a cardio-pulmonary reaction to the tracer and the experiment had to be terminated early (1-hour post tracer administration), while Mouse 2 and Mouse 3 were acquired at 24 hours post tracer administration. The CNR of the site of inflammation was between 8-13 (CNR: contrast-to-noise ratio; SPIO: superparamagnetic iron oxide).

**Figure 8:**
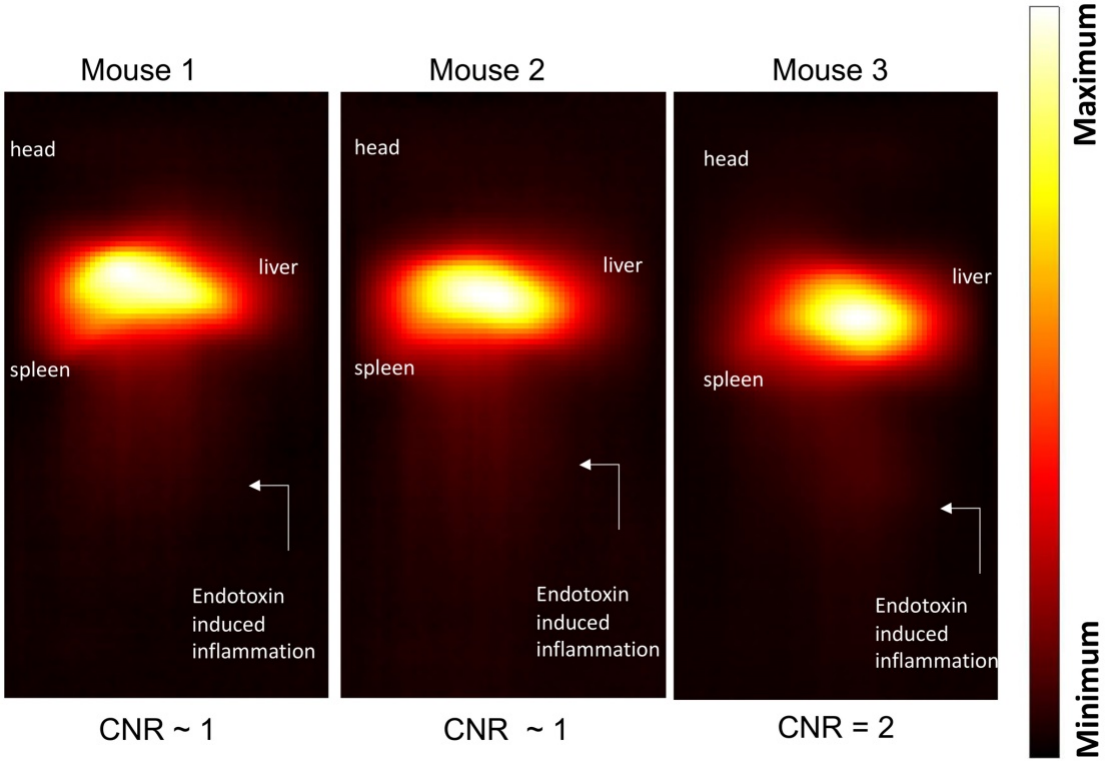
**Inflammation** images in three different mouse subjects with myositis and using **VivoTrax***^TM^* as tracer. At a dose of 5 mg of Fe/kg the CNR was ~1-2 at the site of myositis acquired 24 hours post-tracer administration (CNR: contrast-to-noise ratio).

Maximum-intensity projections (MIP) of 3D MPI images 24 hours post anti-Ly6G SPIO administration are shown in Figure 6. 2D-projection MPI images in three different mice with LPS-induced myositis, and with *i.v.* injections of anti-Ly6G SPIO are shown in Figure 7. Figure 8 shows 2D-projection MPI images of three different mice that with LPS-induced myositis, and with *i.v.* injections of VivoTrax*^TM^*. Contrast-to-noise ratio (CNR) was calculated for each 2D projection by comparing the ROI of the inflamed (i.e. right) flank as compared to the contralateral (i.e. left) flank versus the background noise extracted from the upper left background in each image.

**Figure 9:**
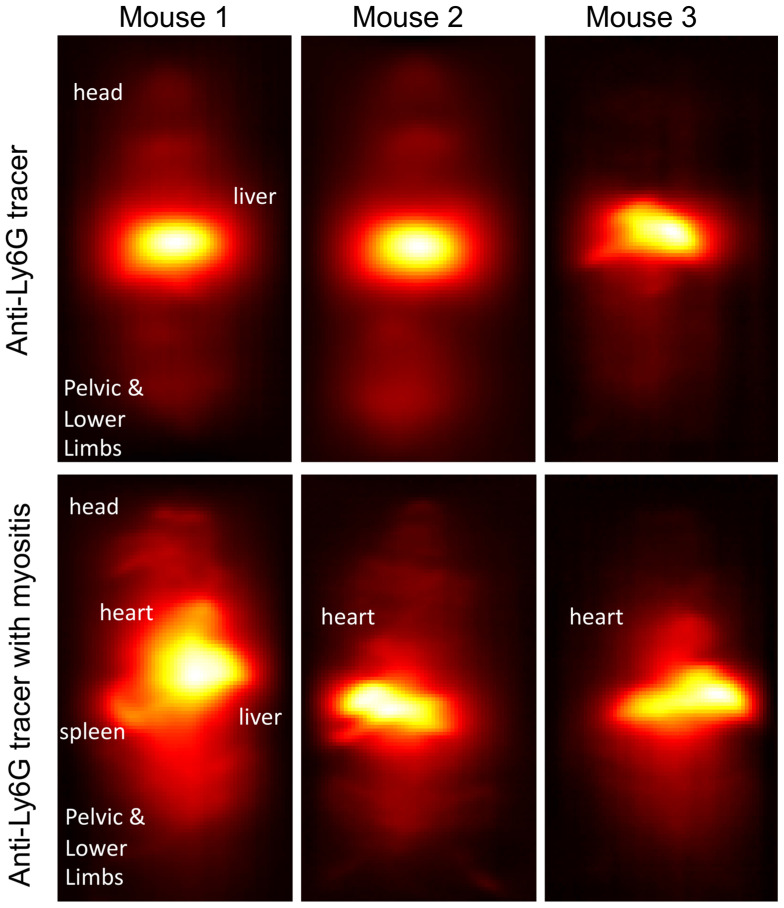
**Change in circulation times of tracer after inducing inflammation**. In healthy mice (top row), anti-Ly6G SPIO tracer predominantly distributes in the organs of the RES including the liver, spleen, and bone marrow, at around 4-5 hours post-tracer administration. We observed a change in the circulation times of the tracer in myositis induced mice (bottom row). These mice showed prominent MPI signal from the ventricles of the heart around 4-5 hours of post-tracer administration indicative of longer circulation times of tracer post inflammation (RES: reticuloendothelial system; SPIO: superparamagnetic iron oxide).

In healthy mice, anti-Ly6G SPIOs rapidly distributed in the RES organs of liver and spleen, and more notably, in the marrows of cranium, limbs and the pelvic bones (Figure 6A & Figure 9 top row). In mice with LPS-induced myositis, the circulation times of *i.v.* injected anti-Ly6G SPIO increased, as observed by prominent signal from the ventricle of the heart (see Figure 9 bottom row). 24 hours after tracer administration, CNRs between 8 and 13 were observed on the inflamed right thigh of anti-Ly6G SPIO administered mice (Figure 6B & 7) as compared to the non-inflamed left thigh.

In comparison, *i.v.* administered VivoTrax*^TM^*, which is coated with carboxydextran, was quickly cleared by the RES system in the circulation and accumulated largely in the liver and to a small extent in the spleen in healthy mice and even in the mice with LPS-induced myositis (Figure 8). This data is in strong agreement with our previous observations 54.

**Figure 10:**
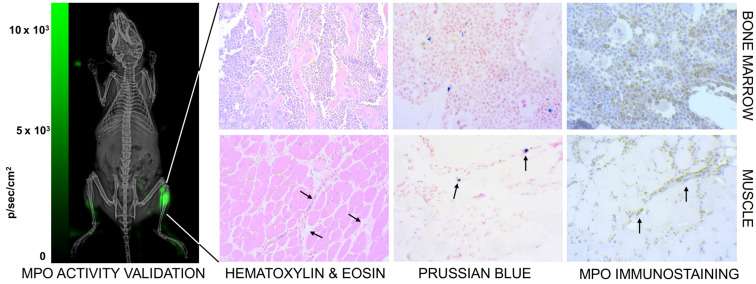
**Confirmation of inflammation.** (Left) The neutrophil activity at the site of myositis was confirmed by using *i.p.* administered luminol, which provides bioluminescent visualization of myeloperoxidase (MPO) enzyme activity. (Right) Histological analysis of anti-Ly6G nanoparticle uptake in mouse models. Images were captured by brightfield microscopy following staining of infected tissues using H&E staining for tissue morphology, Prussian blue staining for SPIO, and immunohistochemistry for myeloperoxidase. The Prussian blue confirmed the distribution of anti-Ly6G SPIO in bone marrow in regions with increased MPO enzyme expression (myeloid cells). The images shown in the bottom row are muscle tissues from the right infected leg. Inflammation injury was observed in the H&E stain (arrows), with the corresponding regions showing areas of increased iron and MPO enzyme activity (H&E: hematoxylin & eosin; MPO: myeloperoxidase; SPIO: superparamagnetic iron oxide (nanoparticles).

We co-validated the MPI images by checking for neutrophil presence using luminol, which is known to luminesce due to neutrophil associated myeloperoxidase activity 78. Figure 10 (left) shows higher bioluminescence from the site of myositis on the right thigh. Further, tissue histology from the inflamed tissue confirmed the localization of anti-Ly6G labeled neutrophils at the site of inflammation, as seen in Figure 10 (right).

The observed difference in CNR for anti-Ly6G particles versus VivoTrax*^TM^* is well supported by existing literature. Intravenously administered nanoparticles in general are known to accumulate in the RES system, including the liver, spleen, and marrow. Combined Sulphur colloids and WBC scintigraphy have been used for diagnosis of osteomyelitis 26. Ultrasmall superparamagnetic iron oxide nanoparticles (USPIOs) with long circulation time and in the size range of 5-15 nm exhibited bone marrow uptake 79. However, colloids the size of VivoTrax*^TM^* had insignificant bone marrow uptake.

Marrows are active centers of the hematopoietic system and have a large pool of myeloid cells expressing Ly6G markers. Imaging with anti-Ly6G SPIOs enabled a marked improvement in contrast of the bone marrow, as seen in Figure 6A & 9 (top row). We attribute this to the antibody binding to the specific cell lineages of the marrow cells, which was also confirmed by Prussian blue staining in the areas of myeloid cells (Figure 10).

When considering the clinical implications of this study, we note that physiological differences do exist between murine models and humans. Under healthy conditions only ~20% of circulating blood cells are neutrophils in mouse, whereas almost ~70% of the circulating white blood cells are neutrophils in humans.

The number of circulating peripheral blood neutrophils in both species does increase at the onset of inflammation 80, which helps explain the increase in circulation time of the administered tracers in inflamed mice. Similar observations were reported with MRI imaging using IgG antibody-SPIO administered in rodents 81. Though that work was done with a polyclonal IgG, with our monoclonal IgG we observed a marked difference in specificity of the administered tracer in terms of their *in vitro* binding and *in vivo* biodistribution with anti-Ly6G SPIO. More detailed work further examining WBC dynamics with different antibody-based SPIOs specific to a greater variety of immune cell such as that of anti-F4/80 SPIOs, will complement this study in the future 44, 82.

## Conclusion

In this work, we harnessed monoclonal antibodies for *in situ* labeling and tracking of neutrophils using MPI. Commercially available libraries of anti-Ly6G SPIOs showed better MPI performance in terms of both signal-to-noise ratio, resolution, and specificity of binding to neutrophils, than the most common MPI tracer, VivoTrax*^TM^*. *In situ* labeled neutrophils showed superior contrast in distinguishing sites of LPS induced myositis. *In situ* tagging of immune cells with anti-Ly6G SPIOs have several advantages compared to *ex vivo* labelling (1) *in situ* labelling reduces the overall preparation time of the study: currently, a clinical WBC study may entail isolating WBCs, *ex vivo* labeling, quality analysis and then tracer administration, and (2) *in situ* labelling reduces the burden on the labeled immune cells: *ex vivo* labeling of immune cells with SPIOs has shown to induce phenotypic changes in the cells 37, and to inhibit proliferation 83. Despite these advantages, several aspects must be considered with the *in situ* labeling approach for tracking WBCs. There are likely to be differences in biodistribution as a result of non-specific tracer binding. Delayed MPI scans for diagnosis, approaches similar to the ones carried out using the clinical In-111 WBC scan, can enable higher specificity as a higher concentration of tracer-labeled cells is expected to accumulate at the site of inflammation over time. Additionally, anaphylactic reactions associated with administering antibodies are a risk. Monoclonal antibodies raised and produced in one organism, when administered to another organism, can result in the production of antibodies against the monoclonal antibody, leading to a toxic response 84, 85. Additionally, mouse models of inflammation using LPS can develop platelet-activating factor (PAF)-mediated anaphylaxis-like shock when the immune cells are stimulated using the administered antibodies 86, resulting in respiratory distress and even death. One approach to mitigate anaphylaxis is to administer antibodies 24-hours post inducing inflammation. Another approach is to engineer antibodies that have negligible Fc receptor binding, which can reduce stimulation of the immune cells. For instance, antibodies with the Fc regions functionalized to a fluorochrome or nanoparticle can significantly mitigate secondary responses 87. Note that monoclonal antibodies are commonly administered to patients as therapeutics, and potential side effects and mitigation strategies are well-understood in humans 88-92.

In summary, we developed a proof-of-concept *in vivo* approach to label and track neutrophils using MPI. This approach used SPIOs conjugated to anti-Ly6G-antibody, which is specific to Ly6G antigen, a Ly6 family of glycosylphosphatidylinositol-linked proteins. Ly6 proteins have been implicated in neutrophil migration and recruitment, and anti-Ly6 antibodies have previously been administered at high concentrations in mouse studies to deplete neutrophils from circulation 93. Moreover, fluorescent probe conjugated antibodies against Ly6 have been used for tracking neutrophils using optical methods 87. We adapted these methods to use anti-Ly6G-conjugated SPIOs to label and track neutrophils *in vivo* using MPI and demonstrated that this method could visualize neutrophil recruitment to inflammation in a mouse model of LPS-induced myositis. Additionally, we characterized commercially available antibody conjugated SPIOs manufactured for cell separation applications and showed that these SPIOs have better MPI performance than a traditional MPI tracer, VivoTrax*^TM^*. Notably, these antibody conjugated SPIOs had better signal and resolution for MPI applications, and higher specificity of binding to neutrophils. Future work using engineered antibodies conjugated to MPI tracer particles will open new avenues for tracking an even larger variety of cell types, with improved specificity and safety profiles. This approach represents a potential alternative to traditional radioactive tracers for tracking WBCs, which can impact the labeled cell phenotype and cause chromosomal aberrations 94, 95. In the future, non-radioactive WBC tracking approaches using MPI and antibody-conjugated SPIOs may be an invaluable tool not only for imaging of inflammation, but also for optimizing and evaluating antibody-based and cell-based immunotherapies.

## Materials and Methods

In all studies, anti-Ly6G-antibody functionalized SPIOs targeting surface antigens expressed in neutrophils were evaluated with VivoTrax*^TM^* as a control. Antibody-conjugated SPIOs were purchased from Miltenyi Biotec GmBH. Anti-F4/80 antibody functionalized SPIOs, specific to surface antigens that are expressed in the tissue residual monocytes, was used as a negative control only for the *in vitro* experiments.

The Miltenyi Biotech beads are particularly useful for MPI, as their superparamagnetic nature (i.e. having zero remanence and high initial susceptibility) resulted in better image resolution and signal than paramagnetic or ferromagnetic equivalents. 1 mL of Ultrapure microbeads of anti-Ly6G REA254.6 IgG1 clone (#130-120-337), and anti-F4/80 IgG1 clone (#130-110-443) were dialyzed before use against sterile 1X phosphate buffered saline overnight with two changes of solvent using a Spectra-Por® Float-A-Lyzer® G2 (MWCO 3.5-5 kDa). The sample was recovered and used as such. The iron content of the particles were estimated colorimetrically using the Perls' Prussian blue reaction 96. In brief, 10 µL of particle sample was mixed with 100 μL of 1.2 mM hydrochloric acid solution (Sigma Aldrich Catalog No. HT202-250 ml) in 96 well black plate with transparent bottom. After 24-hour incubation, 100 µL of 4% w/v potassium ferrocyanide solution was added and incubated at room temperature for thirty-minutes before reading for absorbance at λ = 630 nm using a microplate reader (SpectraMax® i3, Molecular Devices). A standard curve was generated using serial dilution of iron ICP standard (TraceCERT®, Sigma Aldrich (56209)) followed by Perls' Prussian blue assay.

The protein quantification was carried out using classical Comassie Blue (Bradford) protein assay 97. Bright field TEM was performed at 200 keV (Tecnai 12, FEI) to characterize nanoparticle morphology and size. Particle core diameter was calculated from the TEM image using ImageJ software. Hydrodynamic size and Zeta potential of the coated nanoparticles was measured in PBS using Dynamic Light Scattering (DLS, Malvern ZetaSizer Nano ZS). The DC magnetic properties of the particles in solution were measured by Vibrating Sample Magnetometer (7400 Series VSM, Lake Shore Cryotronics, Inc.). Magnetic size was determined by the fitting of M(H) data to a Langevin function assuming a log-normal size distribution (akin to Chantrell's method 58, 98). Specifically, a non-linear least squares fit of the M(H) data was performed to a sum of moments generated by a log-normal distribution of particles with mean diameter *D_v_* in nm and log-normal standard deviation 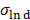 (unitless), with n = 100 logarithmically spaced bins on the interval 3 log-normal standard deviations above and below the mean (i.e. 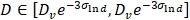). Particles were characterized for MPI performance using our in-house arbitrary wave relaxometer (AWR). The point-spread function and signal were estimated using a DC bias field sweep from -60 mT to 60 mT over 0.3 s and an excitation sinusoidal field of 20 kHz and 20 mT. Each sample was measured 8 times and averaged and had a measurement bin size of 0.1 mT. The characterization for each tracer was performed for 6 serially diluted 40 µl samples with concentration between 0.004-0.125 mg Fe/ml in 1X sterile phosphate buffered saline and in mouse blood (CD-1). Mouse blood was purchased from Lampire Biological Laboratories, Pipersville, PA. Blood was collected and stored in Na-Heparin and was used within two weeks from receiving. For MPI scanner sensitivity, the SPIO tracers were serially diluted from 2.75 - 0.172 mg of Fe/ml in 10 µl tubes in 1X PBS similar to our previous work 2. For blood samples, only the higher Fe concentrations of 1.38 and 0.69 mg of Fe/ml of SPIOs were prepared (as above) in 10µl tubes. 2D-projection MPI scans were acquired with FOV = 4.1 x 6.2 cm^2^, data acquisition time of 37 s, and pixel size = 0.48 x 0.21 mm^2^. The standard curve was then plotted using the average SNR (using the 5 x 5 pixel surrounding the peak). Signal-to-noise ratio (SNR) was estimated as mean of signal over background noise (σ) extracted from the upper left background in each image for each sample. The linear fit of the standard curve for each particle provided the sensitivity in SNR per g Fe/mm^2^, and the limit of detection was then calculated for SNR = 3, with standard deviation derived from the standard deviation in the fit's coefficients.

### Cell Enrichment Analysis of MPI Tracers

To evaluate cell enrichment and to evaluate labeling of neutrophils using antibody based MPI tracer, a qualitative analysis was carried out using transmission electron microscopy. A whole blood sample (Lampire Biological Laboratories, PA) was treated with RBC lysis buffer (Alfa Aesar) to lyse the RBCs following a centrifuged at 300g for 10 minutes. The RBC free pellet was collected and was washed two more times to remove any debris. The cells from the pellet that should contain most of the peripheral WBCs was incubated with 10 µL of anti- Ly6G antibody SPIO particles for an hour in a 36 °C incubator. Following which the pellet was centrifuged twice to remove any unbound SPIOs and fixed using 2.5% glutaraldehyde solution. The pellet was processed for electron microscopy following standard procedure of 1% Osmium tetroxide staining, gelatin fixation, dehydration, araldite embedding, sectioning, and viewing (TEM, Tecnai 12, 120kV TEM, FEI, Hillsboro, OR, USA).

**Figure 11:**
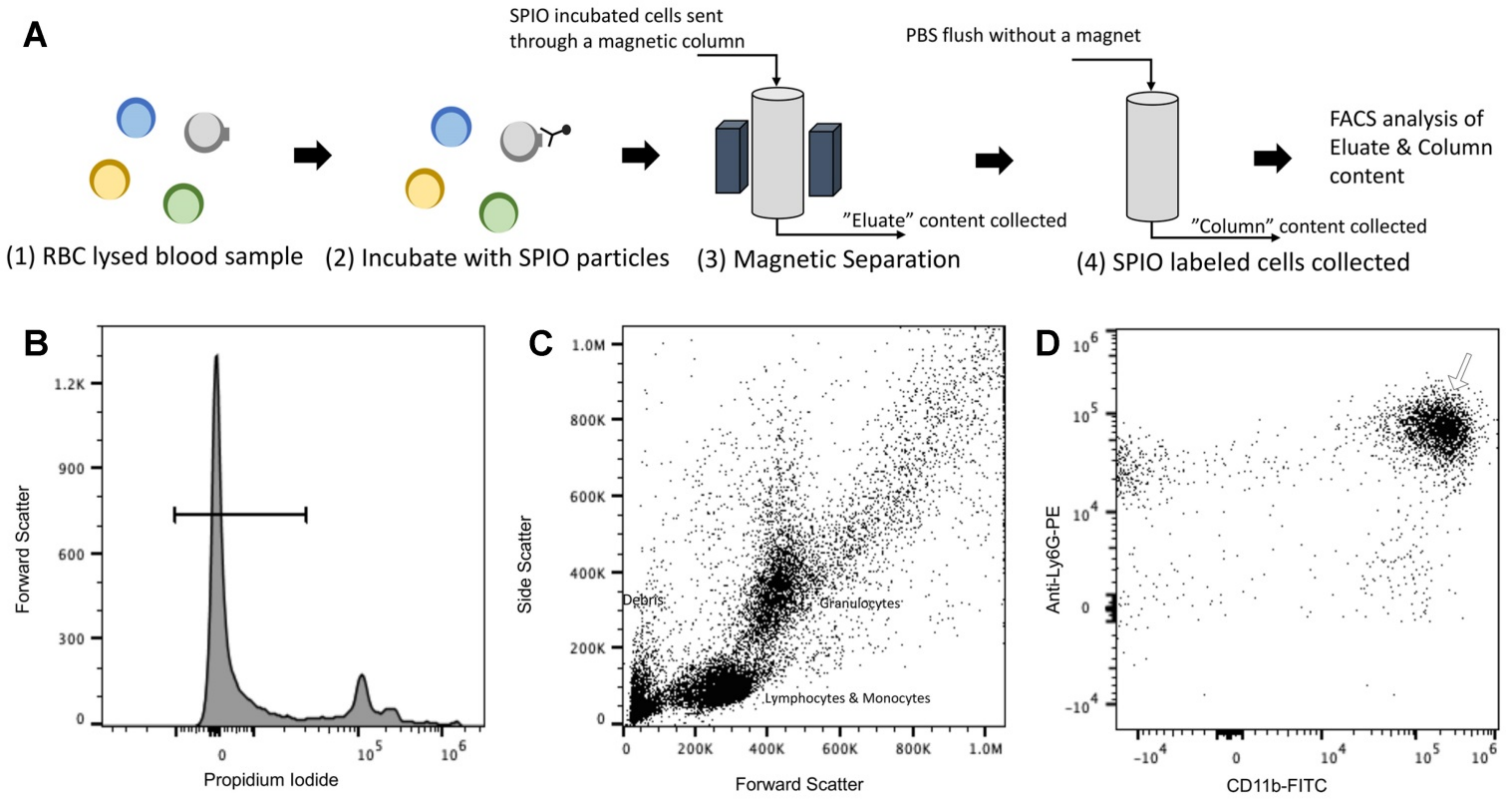
**Cell Enrichment Analysis of MPI tracers using Flow Cytometry** (A) Cells obtained after RBC lysis of mouse blood were incubated with SPIO of interest. The incubated cells were sent through a magnetic column, and unlabeled cells were collected as “eluate” from the column. After removing the magnet utilized for separation, the column was further flushed, and the contents were collected as the “column” subset of cells. The immune cell population from the “eluate” was evaluated for degree of depletion by the SPIO relative to the extent to which it is enriched from the “column” in comparison with control (untouched RBC lysed blood) using flow cytometry. The following gating procedure was carried out on the flow cytometry data: (B) the propidium iodide channel was used to gate live cells, which are negative for the dye, (C) the granulocyte population of immune cells were chosen utilizing the side scatter and forward scatter profile and (D) the cells were analyzed for neutrophil cell marker expression (anti-Ly6G-PE and CD11b-FITC) utilizing the PE and FITC channels. Untouched (i.e. unseparated) samples of RBC lysed blood were used as a control to gauge the native proportion of blood cells in the samples (MPI: magnetic particle imaging; RBC: red blood cells; SPIO: superparamagnetic iron oxide).

The efficiency of antibody based MPI tracers for tagging neutrophils/immune cells was further analyzed using flow cytometry as shown in Figure 11. A known amount of cell pellet, suspended in 1X PBS after RBC lysis of whole blood, was incubated with different MPI tracers (i.e., VivoTrax*^TM^*, anti-Ly6G SPIOs and anti-F4/80 SPIOs). After an hour incubation, the suspension was passed through a magnetic column (Miltenyi Biotec Inc, #130-042-201). The “eluate” was collected under with a magnetic field applied to the column and is formed from the separated untargeted cells (as compared to the particle bound cells in the column). The “column” sample was then collected by flushing the remaining cells in the absence of an applied magnetic field. To emulate conditions similar to *in vivo* experiments, the SPIO labeled cells were only passed once through the magnetic column. Typically, for magnetic enrichment of a particular cell type, the SPIO labeled cells are passed through the column more than once to achieve higher purity. All samples were diluted equally with 1X-PBS and were stained for viability (propidium iodide), and with anti-Ly6G-PE (Miltenyi Biotec Inc, #130-102-392) and CD11b-FITC (Miltenyi Biotec Inc, #130-113-805) fluorophores. The “column” and “eluate” cell sample were measured using an Attune*^TM^* NxT Flow Cytometer and analyzed using FlowJo (BD, Oregon, USA). The gating procedure for analysis of the flow cytometry data is shown in Figure 11. Untreated RBC lysed whole blood was used as control, providing a gauge of the relative population of the blood cells before the enrichment. The population of granulocytes and Ly6G^+^CD11b^+^ cells were calculated as percentage of total counts from the flow cytometer per sample.


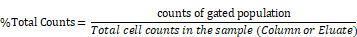


The efficiency of enrichment of granulocytes and Ly6G^+^CD11b^+^ cells in the “column” contents were compared relative to the same population being depleted in the “eluate” content.

### *In vivo* mouse model of myositis

All animal procedures were conducted according to the National Research Council's Guide for the Care and Use of Laboratory Animals and approved by the Animal Care and Use Committee at UC Berkeley. Female C57BL/6 (7-8 weeks old) were obtained from a UC Berkeley Office of Laboratory Animal Care approved vendor. All animals were monitored weekly for behavior and body weight. Inflammation was induced in a C57BL6 mouse by injecting bacterial lipopolysaccharide (LPS) (O111:B4, 50 µg) into the right thigh muscle. Animals were monitored post-LPS administration for any discomfort and difficulties in walking. All LPS treated mice were sacrificed within 48 hours of inducing inflammation.

### Projection, 3D tomography Magnetic Particle Imaging and Image Processing

The bio-distribution was evaluated after *i.v.* administration of VivoTrax*^TM^* and anti-Ly6G SPIO (IgG1, REA526 clone, Miltenyi Biotec, GmbH, after dialysis) iron oxide nanoparticles at a clinically relevant dose (5-5.5 mg of Fe/kg, *∼*40 µg of protein/mouse) using a 6.3 T/m/*µ*_0_ field-free line MPI scanner using 2D projections, with a field-of-view (FOV) of 10.6 × 6.2 cm^2^ and using a 40 mTpp drive field at a frequency of 20.225 kHz.^2^ The scanning bed was mechanically translated in the z-direction in 1 mm increments to complete the imaging trajectory for a single projection, resulting in a five-minute total scan duration, 95 s of which were used for data acquisition. All 2D projection *in vivo* images were taken with respiratory gating, and reconstructed using the x-space MPI reconstruction algorithm (pixel dimension of 0.04 × 0.034 cm^2^) 99, 100. For the inflammation study, the contralateral side of the mouse with respect to the inflamed flank was used as a control. The particles were also injected in a separate cohort of healthy C57BL6 mice. 3D tomography images were acquired post-euthanasia. Forty 2-D projection images were acquired at equally spaced angles and reconstructed using a Radon transformation. All 3D data were re-binned to a voxel dimension of 0.04 × 0.04 × 0.034 cm^3^.

The CNR was estimated from 2D-MPI projection data acquired. All 2D-projection images were scaled between the minimum and maximum value of the image. The CNR was estimated using the mean signal of ROIs from the inflamed (right) and contralateral (left) flank with the standard deviation of the background as follows:


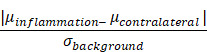


3-D projection images were post-processed using a high-boost filter and represented as a maximum intensity projection (MIPs). All image processing and MPI data analysis was performed using MATLAB (Mathworks, Natick, MA).

### Validation of neutrophil activity and Histology

Bioluminescent validation of localized inflammation was performed by evaluating for myeloperoxidase activity in select mice using an *i.p.* administration of luminol (XenoLight RediJect Chemiluminescent Inflammation Probe, PerkinElmer, Waltham, MA). 100 µL of luminol was injected 24-hours post LPS-induced myositis, and bioluminescence was measured 10-min after the *i.p.* injection, using an IVIS Lumina system with a five-minute exposure time and medium binning. The bioluminescent images were analyzed with Fiji 101 and co-registered with an X-ray projection image, taken with XPERT cabinet X-ray system (KUB Technologies, Inc.), as anatomical reference. Tissue were harvested after euthanization and fixed in 10% formalin. Fixed tissue sample was stained for Prussian blue, hematoxylin and eosin (H&E), and myeloperoxidase (LifeSpanBio Sciences, Inc.). Bright field images of the slides were observed using a ZEISS AX10 Observer D1 with a ZEISS Axiocam ERc 5s (ZEISS Microscopy, Germany).

All statistical analysis was carried out using standard student t-test of the means.

## Figures and Tables

**Table 1:** Physical and MPI properties of evaluated tracers

Tracer Name	Core Size (nm)	D_H_^#^ (nm)	PDI	Zeta (mV)	Resolution* (mm)	LoD*^†^* (ng of Fe)
Anti-Ly6G SPIO	14.0 ± 2	78	0.097	-12.2 ± 0.6	1.26	13 ± 1
VivoTrax^TM^	5.4 ± 1	61	0.223	-12.7 ± 1.8	1.49	22 ± 3

*The measurements reported were carried out using an arbitrary wave relaxometer (AWR) and are converted for a 7 T/m gradient field-free-line MPI scanner operating at 20 mT and 20 kHz excitation. ***^†^***Calculations for the limit of detection (LoD) were based on a standard curve taken in the scanner (Figure 3, FOV = 4.1 x 6.2 cm^2^, scan time = 37 s) estimated for a pixel size of 1.4 × 1.4 mm^2^. Standard deviation is propagated from the error in the linear fitting of the curve. #Hydrodynamic diameter and zeta potential measured using Nano Zetasizer (D_H_: Hydrodynamic diameter; FOV: field-of-view; LoD: Limit of detection; PDI: polydispersity index).

**Table 2:** Magnetic characteristics of evaluated tracers

Tracer Name	M_sat_^#^ (emu/g Fe)	<H_sat_>*^†^* (mT)	D_v_^*^ (nm)	σ_ln d_^*^ (unitless, logscale)
Anti-Ly6G SPIO	44 ± 3	4.4	14.8	0.5
VivoTrax*^TM^*	131 ± 1	5.2	9.8	0.2

# At ±1 T, Lake Shore Cryotronics 7400 series Vibrating Sample Magnetometer. ***^†^***Value derived from the full width at half maximum (FWHM) of the point spread function of the particle in an Arbitrary Wave Relaxometer. * Mean values derived from fit of M(H) curve to the ensemble Langevin from a log-normal distribution of spherical magnetite particles at 290 K (D_v_: mean magnetic diameter; H_sat_: saturating field; FWHM: full width at half maximum; M_sat_: saturation magnetization; mT: milli-tesla; nm: nanometer).

## Abbreviations

2D: two-dimension
3D: three-dimension
AWR: arbitrary wave relaxometer
CT: computed tomography
CNR: contrast-to-noise ratio
D_H_: Hydrodynamic diameter
DLS: dynamic light scattering
D_v_: mean magnetic diameter
FITC: Fluorescein isothiocyanate
FOV: field-of-view
FFL: field-free line
FUO: fever of unknown origin
GI: gastrointestinal
H: magnetic field
H&E: Hematoxylin and eosin
H_sat_: saturating field
i.p.: Intraperitoneal injection
i.v.: intravenous
LoD: Limit of detection
LPS: lipopolysaccharides
PDI: polydispersity index
PE: Phycoerythrin
M: Magnetization
MIP: maximum intensity projection
MPI: magnetic particle imaging
MPO: myeloperoxidase
MRI: magnetic resonance imaging
M_sat_: saturation magnetization
mT: millitesla
NCA: non-specific cross-reacting antigen
nm: nanometer
PAF: platelet activation factor
PBS: phosphate buffered saline
PI: propidium iodide
PSF: point-spread function
RBC: red blood cells
RES: reticuloendothelial system
ROI: region of interest
SNR: signal-to-noise ratio
SPIO: superparamagnetic iron oxide (nanoparticles)
SSTR: somatostatin receptors
TEM: transmission electron microscopy
VSM: vibrating sample magnetometer
VT: VivoTrax^TM^
WBC: white blood cells
